# Economic burden and financial vulnerability of injuries among the elderly in Vietnam

**DOI:** 10.1038/s41598-023-46662-3

**Published:** 2023-11-07

**Authors:** Hai Minh Vu, Linh Gia Vu, Tung Hoang Tran, Laurent Boyer, Pascal Auquier, Guillaume Fond, Tham Thi Nguyen, Linh Khanh Le, Hoa Thi Do, Huyen Phuc Do, Son Nghiem, Carl A. Latkin, Roger C. M. Ho, Cyrus S. H. Ho

**Affiliations:** 1 Department of Trauma, Thai Binh University of Medicine and Pharmacy, Thai Binh, 410000 Vietnam; 2 Institute for Global Health Innovations, Duy Tan University, Da Nang, 550000 Vietnam; 3 Faculty of Medicine, Duy Tan University, Da Nang, 550000 Vietnam; 4 Institute of Orthopedic and Trauma Surgery, Vietnam – Germany Hospital, Hanoi, 100000 Vietnam; 5 Research Centre on Health Services and Quality of Life, Aix Marseille University, 27, Boulevard Jean-Moulin, 13385 Marseille Cedex 05, France; 6 Troy University, Troy, AL 36082 USA; 7 Institute of Health Economics and Technology (iHEAT), Hanoi, 100000 Vietnam; 8 Centre for Applied Health Economics, Griffith University, Brisbane, QLD 4111 Australia; 9 Bloomberg School of Public Health, Johns Hopkins University, Baltimore, MD 21205 USA; 10 Department of Psychological Medicine, Yong Loo Lin School of Medicine, National University of Singapore, Singapore, 119228 Singapore; 11 Institute for Health Innovation and Technology (iHealthtech), National University of Singapore, Singapore, 119077 Singapore

**Keywords:** Health care, Medical research

## Abstract

Falls are a leading cause of death among elderly people. However, research on the cost of fall-related injuries is limited in Vietnam. We estimated treatment costs and associated factors among 405 elderly patients in Thai Binh hospitals. Costs were estimated through self-reported data on medical and non-medical expenses. Logistic regression and GLM were used to identify payment and affordability factors. Inpatient and outpatient care costs for fall-related injuries were US$98.06 and US$8.53, respectively. 11.85% of participants couldn’t pay for treatment. Payment ability and cost decline were linked to family income, medical history, and hospital stay length. Elderly with fall-related injuries in Vietnam experienced high costs and severe health issues. Primary healthcare services and communication campaigns should be strengthened to reduce disease burden and develop effective fall injury prevention strategies.

## Introduction

Falls and fall-related injuries are a significant health concern for older adults, with the World Health Organization defining a fall as “a state of lying on the ground or floor or other low levels caused by unintentional activities”^1,2^. The incidence of falls is increasing as the world's population ages, with higher rates observed in developing countries compared to industrialized nations^3,4^. Globally, an estimated 33% of older adults experience at least one fall each year, with approximately half requiring medical attention due to multiple falls^4,5^. Falls are the leading cause of injury and mortality among those aged over 65, and the World Health Organization predicts that falls could rise from 21st to 17th place in the worldwide ranking of causes of mortality by 2030 if preventative measures are not implemented^6^. Falls not only increase the risk and severity of injuries and related diseases in the elderly, but also lead to significant disability, dysfunction, and death, resulting in higher treatment costs and lower quality of life^7,8^.

Research has attempted to quantify the burden of fall injuries, with those requiring medical attention being more likely to have comorbidities than those without falls. Comorbidity is associated with reduced recovery, increased risk of long-term disability, and mortality^9–14^. Fall-related injuries also place a significant economic burden on patients and their families due to the high cost of treatment^15^. In Vietnam, the average cost of treating fall-related injuries is approximately US $145, with additional expenses such as meals, transportation, and caregiving also adding to the financial burden^16^.

Vietnam's aging population has been increasing rapidly, with older adults accounting for 11.8% of the total population in 2019, compared to 8.7% in 2009^17^. Given this trend, developing effective fall prevention, treatment, and care strategies for the elderly is crucial. Although several studies have investigated the costs of falls, in Vietnam, studies focused on treatment costs and the impacts of falls on older adults have not been thoroughly investigated. Therefore, this study aims to estimate the treatment costs and cost burdens of elderly patients hospitalized for falls and investigate factors related to the costs of falls, filling the gaps in the literature^18–20^.

## Results

Table 1 compares the covariates between inpatients and outpatients in Thai Binh Hospital. Among 405 patients, most of them lived in rural areas (92.10%), were female (60.00%), lived with spouses/partners (67.65%), and had a caregiver (95.80%). 349 patients accounting for more than 80.00% of the population had a high school education or lower. Reportedly, 97.53% were covered with health insurance. The median age of this population was 70 years old, with the interquartile range from 65 to 78. The family monthly income of patients was US $ 255.80 (Interquartile Range-IQR = 170.53–341.06) and the poorest income quintiles accounted for 20.0% of participants. Statistically significant differences between inpatient and outpatient participants were reported in education, household income quintiles, and age (*p* < 0.05).

**Table 1:** Comparison of socioeconomic status and behavior between inpatient and outpatient.

Characteristics	Type of patient				Total		*p*-value
	Inpatient		Outpatient				
	n	%	n	%	n	%	
Total	152	37.53	253	62.47	405	100.00	
Living area							
Urban	18	11.84	14	5.53	32	7.90	0.023
Rural	134	88.16	239	94.47	373	92.10	
Gender							
Male	61	40.13	101	39.92	162	40.00	0.967
Female	91	59.87	152	60.08	243	60.00	
Education							
Not going to school	28	18.42	21	8.30	49	12.10	< 0.001
Primary school	50	32.89	92	36.36	142	35.06	
High school	45	29.61	113	44.66	158	39.01	
Above high school	29	19.08	27	10.67	56	13.83	
Marital status							
Single/Divorce/Widow	53	34.87	78	30.83	131	32.35	0.400
Living with spouse/partner	99	65.13	175	69.17	274	67.65	
Caregiver							
No	7	4.61	10	3.95	17	4.20	0.751
Yes	145	95.39	243	96.05	388	95.80	
Having health insurance							
No	4	2.63	6	2.37	10	2.47	1.000
Yes	148	97.37	247	97.63	395	97.53	
Monthly household income quintiles							
Poorest	41	26.97	40	15.81	81	20.00	< 0.001
Poor	29	19.08	80	31.62	109	26.91	
Normal	11	7.24	44	17.39	55	13.58	
Rich	25	16.45	62	24.51	87	21.48	
Richest	46	30.26	27	10.67	73	18.02	

	Median	IQR	Median	IQR	Median	IQR	*p*-value
Age (Unit: years)	71.5	65.0–80.0	69.0	64.0–77.0	70.0	65.0–78.0	0.023
Monthly household income (unit: US$)	255.80	127.90–426.33	255.80	170.53–298.43	255.80	170.53–341.06	0.182

The treatment and its characteristics reported by the participants were presented in Table 2. Hypertension (34.07%), Skeleton/Cartilage problems (32.84%), and Spine problems(21.73%) were the most common health issues among elderly patients. 58.54% were reported falling more than once in the last 12 months. There was a significant difference between inpatient and outpatient in the types of injuries-related falls (*p* < 0.05), with the number of hard-tissue injury cases, being higher in the inpatient group (90.79%) than in the outpatient group (45.85%). Additionally, 11.85% of participants reported that they were unable to afford the cost of treatment.

**Table 2:** The characteristics of treatment and illness of the participants.

Characteristics	Type of patient				Total		*p*-value
	Inpatient		Outpatient				
	n	%	n	%	n	%	
History of health issues							
Hypertension	66	43.42	72	28.46	138	34.07	0.002
Cardiovascular	22	14.47	31	12.25	53	13.09	0.521
Ear problems	10	6.58	5	1.98	15	3.70	0.018
Spine problems	25	16.45	63	24.90	88	21.73	0.046
Skeleton/Cartilage problem	41	26.97	92	36.36	133	32.84	0.051
Others	40	26.32	38	15.02	78	19.26	0.005
Number of falling							
Once	34	57.63	34	32.38	68	41.46	0.002
More than once	25	42.37	71	67.62	96	58.54	
Type of current fall injuries							
Soft tissue injuries	14	9.21	137	54.15	151	37.28	< 0.001
Hard tissue injuries	138	90.79	116	45.85	254	62.72	
Ability to afford the payment							
Unable	36	23.68	12	4.74	48	11.85	< 0.001
Partially	60	39.47	49	19.37	109	26.91	
Completely	56	36.84	192	75.89	248	61.23	

	Mean	SD	Mean	SD	Mean	SD	*p*-value
Duration of hospitalization (Unit: days)	7.95	3.59	1.06	0.55	3.65	4.02	< 0.001

Table 3 presents the medical cost of the participants. In general, inpatient payment (median = US $ 98.06, IQR = 56.66; 170.52) was higher in comparison with the payment of outpatients (median = US $ 8.53, IQR = 6.39; 14.84). Travel and food were the two main components contributing to the non-medical expenses. The total non-medical cost for the inpatient group was US $ 76.74 (IQR = 42.21; 102.32), and for the outpatient group was US $ 6.39 (IQR = 5.54; 12.79). Regarding the direct medical cost, the median for the hospitalized patients was US $ 47.37 (IQR = 21.65; 111.67), versus US $ 3.98 (IQR = 2.11; 5.63) for the other group. The results also showed that surgery cost was the major component of direct medical cost, accounting for 79.19% (median = US $ 37.52; IQR = 4.16; 85.27) and 67.95% (median = US $ 2.70; IQR = 1.86; 3.29) of the total cost in inpatient and outpatient groups, respectively.

**Table 3:** The medical cost of treatment among patients with fall injury. Unit: US $

Characteristics	Type of patient							
	Inpatient				Outpatient			
	n	Median	IQR	%	n	Median	IQR	%
Direct non-medical cost								
Travel	151	17.05	11.94–25.58	22.22	248	4.26	2.13–8.53	66.67
Food	151	51.16	26.86–72.48	66.67	230	4.26	2.13–6.39	66.67
Total non-medical cost	152	76.74	42.21–102.32	100.00	248	6.39	5.54–12.79	100.00
Direct medical cost								
Health examination	102	0.32	0.28–0.33	0.67	38	0.30	0.26–1.28	7.50
Medication	101	5.12	2.56–11.94	10.80	32	1.28	0.66–2.22	32.15
Lab test	102	8.53	5.12–12.79	18.00	36	1.06	0.53–2.64	26.58
Surgery	75	37.52	4.16–85.27	79.19	18	2.70	1.86–3.29	67.95
Bed	101	15.35	10.23–21.32	32.40	0		–	–
Others	26	0.59	0.43–2.98	1.24	2	2.00	1.02–2.98	50.38
Total direct medical cost	102	47.37	21.65–111.67	100.00	38	3.98	2.11–5.63	100.00
Total medical cost (unit: US$)	152	98.06	56.66–170.52	92.00	249	8.53	6.39–14.84	8.00

*n* number of patients responding to the questions, % Percentage by unit cost, *IQR* interquartile range.

Table 4 and Appendix S1 shows the associated factors related to the ability to afford the payment and the total medical cost. The patients living with their spouse/partner had a higher capacity to pay than those living alone (OR = 2.14, 95% CI = 1.22; 3.76). Education level was associated with a high ability to afford the cost of care. Compared to inpatient participants, outpatients were found to be significantly correlated with a higher ability to afford the costs and lower cost of medicine. The medical cost for fall patients who had a caregiver was lower than for those who did not have one (Coef. = − 1.11, 95% CI = − 1.56; -0.66). Patients who had hard-tissue injuries paid significantly more than those having soft-tissue injuries (Coef. = 1.15, 95% CI = 0.32; 1.98).

**Table 4:** The cost of illness and factors associated with the capacity to afford payment.

Characteristics	Ability to afford the payment		Total medical cost	
	OR	95% CI	Coef.	95% CI
Individual characteristics				
Gender (vs Male)				
Female			0.32*	− 0.03; 0.66
Marital status (vs Single/Divorce/Widow)				
Living with spouse/partner	2.14***	1.22; 3.76		
Caregiver (vs No)				
Yes			− 1.11***	− 1.56; − 0.66
Monthly household income quintiles (vs Poorest)				
Poor	2.29**	1.09; 4.80		
Normal	1.04	0.45; 2.41		
Rich	1.03	0.49; 2.16		
Richest	1.27	0.60; 2.70		
Education (vs Not going to school)				
Primary school	2.38**	1.05; 5.38		
High school	4.33***	1.84; 10.19		
Above high school	3.80***	1.42; 10.17		
Health status				
Type of patients (vs Inpatient)				
Outpatient	2.47***	1.24; 4.91	− 2.37***	− 2.63; − 2.11
Type of current fall injuries (vs Soft tissue injuries)				
Hard tissue injuries	0.63	0.36; 1.11	1.15***	0.32; 1.98
History of health issues (Yes vs No)				
Hypertension	0.70	0.43; 1.16		
Spine problems	1.54	0.83; 2.85	0.50**	0.12; 0.89
Others	0.49**	0.27; 0.90		
Total medical cost (unit: US $)	1.00*	0.99; 1.00		

***p < 0.01, **p < 0.05, *p < 0.1.

## Discussion

In this study, the total cost of post-fall treatment for inpatients was found to be US $98 on average, while for outpatients, it was US $8.5. It also demonstrated associations among the median household income, type of fall injuries, and history of health issues with the patient's ability to pay for the cost associated with falls.

Compared to other studies on the same topic, the fall treatment costs of patients in our study were lower^18,21^. This can be explained by disparities in medical expenses between nations as well as differences in healthcare quality. Measures to aid in fall prevention can help to reduce this unjustified cost burden^1^. Similar to previous studies, our study found that direct non-medical costs, including food and travel costs, accounted for most of the total costs that patients must pay^22,23^. However, in some other studies on the same topic, direct medical costs accounted for the highest proportion of patients, being higher than direct non-medical costs. This difference can be explained by the direct cost estimation method used in those studies^15^. Surgical charges constituted the greatest share of hospitalization costs for older patients with fall injuries, with patients with a history of low back pain or hard-tissue injuries paying more than other patients^1^. The higher treatment costs and lower affordability for inpatients compared to outpatients reflected the fact that patients tended to prefer inpatient medical services, resulting in higher administrative costs. To reduce the economic burden on patients with fall injuries, there is a need to enhance high-value medical supply management, regulate medical equipment providers' excessive profits, and fight for cost-effective material selection without sacrificing patient safety^1^.

Our study found that falls patients who live with spouses/partners can afford treatment costs higher than patients who live alone. Besides, when there was a caregiver, patients treated after a fall would have to pay less than other patients. Having spouses or partners can play a critical role in assisting with daily activities, mobility, rehabilitation exercises, or even emotional and psychological support, which is crucial for patients recovering from fall-related injury, ensuring that patients can recover more effectively. Furthermore, the findings in our study show that family members have a vital role in lowering healthcare costs. Spouses or partners can assist in reducing healthcare bills and insurance claims, as well as ensuring that the patient's medical needs are satisfied. Their presence can also reduce the financial burden by perhaps eliminating the need for pricey professional caregivers or lengthier hospital stays. This research emphasizes the value of splitting treatment costs for falling patients who live with spouses/partners. The presence of a spouse or partner offers a sense of financial security and alleviates concerns about paying treatment fees, which can greatly reduce the financial pressure associated with medical treatments^15,24^. This finding suggests that besides fall-prevented programs, caregiver training and support programs can be established to empower spouses/partners to provide effective care and support at home, reducing the need for extensive hospital stays or costly professional assistance.

Some limitations of this study should be acknowledged. The convenience sampling used in the study does not reflect representative results for all patients treated after a fall in Vietnam. Additionally, for a cross-sectional study, information about the health status or expenditure was collected from patients' self-reports, which might lead to recall errors in cost measurement. Numerous indirect expenditures, such as healthcare personnel, fixed assets, administration, and equipment operating fees, were excluded from the projected cost provided in this study. These expenses were removed owing to time and resource constraints, but they deserve additional future research.

## Conclusion

The results of this study show that fall injuries pose a significant financial burden to the elderly, especially to those who live alone and patients with low incomes. Fall prevention programs are essential to reduce the risk of falls, and the financial burden on the patient’s family and society. In addition, caregivers and medical staff should be propagated and instructed on how to prevent falls, especially when taking care of patients with chronic conditions such as stroke and high blood pressure to improve their knowledge of fall prevention. Fall prevention for the elderly will not only relieve people from the economic burden but also improve their health outcomes.

## Methods

### Study design and sample

A cross-sectional study was conducted from August 2018 to February 2019 in seven hospitals of Thai Binh province, including Thai Binh Provincial General Hospital and six district hospitals (Kien Xuong, Quynh Phu, Tien Hai, Thai Thuy, Dong Hung, and Hung Ha). The selection criteria for participants included (1) 60 years of age and above; (2) Hospitalization (inpatient and outpatient) due to a fall; (3) No cognitive impairment. Participants who suffered from severe injuries and were not able to answer the questionnaire were excluded from the study. Respondents were recruited using the convenience sampling method. A total of 430 patients joined this study with a response rate of 94.2%.

### Measurement and instrument

Older patients were introduced briefly to the study’s purpose, as well as their benefits and rights while participating. After asking them to sign a written informed consent, thirty-minute face-to-face interviews were performed via a structured questionnaire by trained undergraduate medical students from the Thai Binh University of Medicine and Pharmacy. The collected information from the structured questionnaire is described below:

#### Primary outcome

##### Cost of fall injury treatment

Cost per inpatient and outpatient visit were computed by asking the patients to report their expenditure on fall injuries and look at their hospital bills after fall treatment. Data collectors helped patients list all the cost components for fall-injury treatment. The patients then estimated the costs for each activity. The unit costs comprised of two categories (1) direct medical costs (health examination, medication, lab test, hospitalization cost, surgery, and other direct medical costs) and (2) direct non-medical costs (travel and food expenses)^25^.

The total cost of fall injury treatment was calculated by summing the total cost for direct medical care after deducting the covered health insurance and other direct non-medical costs. All cost data were obtained in Vietnamese Dong (VND), and the final values were given in US dollars, with 23,465 VND equaling 1 US dollar at the 2019 conversion cost.

##### Ability to afford the expenditure

We estimated the ability to pay by asking patients to report whether they fully or partially paid or were unable to pay for the fall injury treatment.

#### Predictor variables

##### Social demographic

We asked participants to self-report their Living area (Urban/Rural), Age, Gender (Male/Female), Level of education (No school/Primary school/High school/Above high school), Marital status (Single, Divorced, Widow/Living with a spouse or partner), Having a caregiver (Yes/No), Monthly household income. In addition, we asked the participants whether they had health insurance.

##### Treatment and illness

The participants self-reported some clinical indicators, such as Type of patient (Inpatient/Outpatient), History of health issues (Hypertension/Cardiovascular/Ear problems/Spine problems/Skelton or Cartilage problem/Other), Number of falls (Once/More than once), Type of current fall injuries (Soft tissue injuries/Hard tissue injuries), Duration of hospitalization.

### Data analysis

STATA version 14.0 (Stata Corp. LP, College Station, United States of America) was utilized to analyze the collected data. For descriptive analysis, the Chi-square test and Man-Whitney test were used to compare the difference of various covariates (Social-demographic and Treatment and illness) among the inpatient and outpatient participants. Generalized linear models, with Gaussian family and identity-link, and Logistic regression model were used to identify the associated factors related to total payment and the ability to afford the expenditure. We utilized a forward stepwise selection strategy, which included variables having a *p*-value of < 0.2 of the log-likelihood ratios tests, along with regression models to construct the reduced model. A *p*-value of less than 0.05 was statistically significant.

### Ethics approval and consent to participate

All participants have explained the objectives of the research and received a written consent form. The study protocol was approved by the Institutional Review Board of Thai Binh University of Medicine and Pharmacy (Code: 7641/HDDD) and performed according to the Helsinki declaration guideline. Informed written consent was obtained from all participants. Participants could refuse to participate at any time without any impact on their treatments. Their data was kept in safe places, and only the principal investigators could access the data.

### Supplementary Information

Supplementary Table S1.

## Data Availability

The datasets used and/or analyzed during the current study are available from the corresponding author upon reasonable request.
