# Supplementary material for: Economic burden and financial vulnerability of injuries among the elderly in Vietnam
Source: Sci Rep. 2023 Nov 7;13:19254. doi: 10.1038/s41598-023-46662-3 (PMC10630303; doi:10.1038/s41598-023-46662-3)
Supplement: Supplementary file 1 — Supplementary Table S1. [file 41598_2023_46662_MOESM1_ESM.docx]

**APPENDIX**

**Tabel S1.** The cost of illness and factors associated with the capacity to afford payment (full model)

| **Characteristics** | **Ability to afford the payment** (n=401) | | **Total medical cost** (n=401) | |
| --- | --- | --- | --- | --- |
|  | **OR** | **95% CI** | **Coef.** | **95% CI** |
| **INDIVIDUAL CHARACTERISTICS** |  |  |  |  |
| **Gender** (vs Male) | 0.77 | 0.45; 1.33 | 0.31 | -0.17; 0.79 |
| Female |  |  |  |  |
| **Marital status** (vs Single/Divorce/Widow) |  |  |  |  |
| Living with spouse/partner | 2.04** | 1.10; 3.80 | -0.23 | -0.83; 0.36 |
| **Caregiver** (vs No) |  |  |  |  |
| Yes | 0.84 | 0.19; 3.64 | -0.96** | -1.82; -0.10 |
| **Living area** (vs Urban) |  |  |  |  |
| Rural | 0.56 | 0.22; 1.40 | -0.24 | -0.66; 0.18 |
| **Having health insurance** (vs No) |  |  |  |  |
| Yes | 1.98 | 0.41; 9.55 | -0.21 | -1.11; 0.68 |
| **Monthly household income quintiles** (vs Poorest) |  |  |  |  |
| Poor | 2.23** | 1.04; 4.76 | 0.35 | -0.25; 0.95 |
| Normal | 0.95 | 0.40; 2.27 | 0.05 | -0.71; 0.81 |
| Rich | 0.99 | 0.45; 2.17 | -0.55 | -1.28; 0.17 |
| Richest | 1.24 | 0.56; 2.74 | -0.12 | -0.56; 0.33 |
| **Education** (vs Not go to school) |  |  |  |  |
| Primary school | 2.37** | 1.04; 5.42 | -0.44 | -1.10; 0.22 |
| High school | 3.96*** | 1.65; 9.53 | -0.35 | -0.94; 0.23 |
| Above high school | 3.20** | 1.14; 8.93 | -0.00 | -0.88; 0.87 |
| **HEALTH STATUS** |  |  |  |  |
| **Type of patients** (vs Inpatient) |  |  |  |  |
| Outpatient | 2.06 | 0.84; 5.04 | -1.56*** | -2.56; -0.57 |
| **Type of current fall injuries** (vs Soft tissue injuries) |  |  |  |  |
| Hard tissue injuries | 0.60* | 0.34; 1.08 | 0.70 | -0.38; 1.78 |
| **History of health issues** (Yes vs No) |  |  |  |  |
| Hypertension | 0.72 | 0.43; 1.21 | 0.38 | -0.29; 1.04 |
| Cardiovascular | 0.82 | 0.41; 1.65 | -0.24 | -1.00; 0.52 |
| Ear problems | 0.63 | 0.17; 2.33 | 1.19** | 0.17; 2.21 |
| Spine problems | 1.73* | 0.90; 3.31 | 0.46 | -0.39; 1.32 |
| Skeleton/ Cartilage problem | 0.77 | 0.45; 1.34 | -0.42 | -1.11; 0.28 |
| Others | 0.45** | 0.24; 0.84 | 0.18 | -0.43; 0.78 |
| **Duration of hospitalization** (Unit: days) | 0.95 | 0.84; 1.07 | 0.13*** | 0.07; 0.18 |
| **Total medical cost** (unit: US $) | 1.00 | 0.99; 1.00 |  |  |
| **Ability to afford the payment** (vs Unable) |  |  |  |  |
| Able |  |  | -0.02 | -0.42; 0.39 |
| *** p<0.01, ** p<0.05, * p<0.1 |  |  |  |  |
